# The Proteomics Big Challenge for Biomarkers and New Drug-Targets Discovery

**DOI:** 10.3390/ijms131113926

**Published:** 2012-10-29

**Authors:** Rocco Savino, Sergio Paduano, Mariaimmacolata Preianò, Rosa Terracciano

**Affiliations:** Department of Health Sciences, Laboratory of Mass Spectrometry and Proteomics, University “Magna Græcia”, Catanzaro, University Campus, Europa Avenue, 88100 Catanzaro, Italy; E-Mails: savino@unicz.it (R.S.); paduano@unicz.it (S.P.); preiano@alice.it (M.P.)

**Keywords:** biomarkers, mass spectrometry, MALDI-TOF, proteomics, bodily fluids, tissues

## Abstract

In the modern process of drug discovery, clinical, functional and chemical proteomics can converge and integrate synergies. Functional proteomics explores and elucidates the components of pathways and their interactions which, when deregulated, lead to a disease condition. This knowledge allows the design of strategies to target multiple pathways with combinations of pathway-specific drugs, which might increase chances of success and reduce the occurrence of drug resistance. Chemical proteomics, by analyzing the drug interactome, strongly contributes to accelerate the process of new druggable targets discovery. In the research area of clinical proteomics, proteome and peptidome mass spectrometry-profiling of human bodily fluid (plasma, serum, urine and so on), as well as of tissue and of cells, represents a promising tool for novel biomarker and eventually new druggable targets discovery. In the present review we provide a survey of current strategies of functional, chemical and clinical proteomics. Major issues will be presented for proteomic technologies used for the discovery of biomarkers for early disease diagnosis and identification of new drug targets.

## 1. Introduction

Over the past few years, mass spectrometry (MS)-based proteomics has expanded its interface role to the broad and diverse research areas of science and technology [1–3]. Tremendous progress in MS instrumentation has extended the sensitivity, accuracy and speed of analysis enabling the identification of thousands of proteins per experiment [1,4]. Beyond identification, MS has also greatly implemented quantitation issues [5]. These efforts have provided a powerful tool to assess qualitative-quantitative differences in protein profiles of different samples, in particular diseased *vs.* normal. Proteomic approaches have been increasingly applied to the study of clinical samples, such as cell lysates, tissues or body fluids, with the purpose of discovering novel disease-specific protein biomarkers.

The application of proteomics to the study of human diseases and translation of this technology to the clinic has lead to a new field defined as clinical proteomics [6]. By assessing protein expression profiles and post-translational modifications (PTMs) in healthy and diseased, or drug-treated samples, clinical proteomics has the potential to discover, identify and quantify novel biomarkers to facilitate the early detection, diagnosis and therapeutic intervention of disease. Found as “needles in the haystack” of complex proteome, these biomarkers, as molecular targets, could provide valuable information for drug discovery.

In addition to drug target identification, MS-based proteomics can be employed to accelerate several different steps of the drug discovery pipeline. The power of MS-based functional proteomics has allowed the characterization of cellular, subcellular or organismal proteins providing significant insight into cell biological processes and signal transduction pathways which are at the basis of the drug discovery [7–9].

Recent developments in chemical proteomics have enabled a more direct and unbiased analysis of a drug’s mechanism of action in the context of the proteome as expressed in the target cell or the tissue of interest [10].

**Figure 1:**
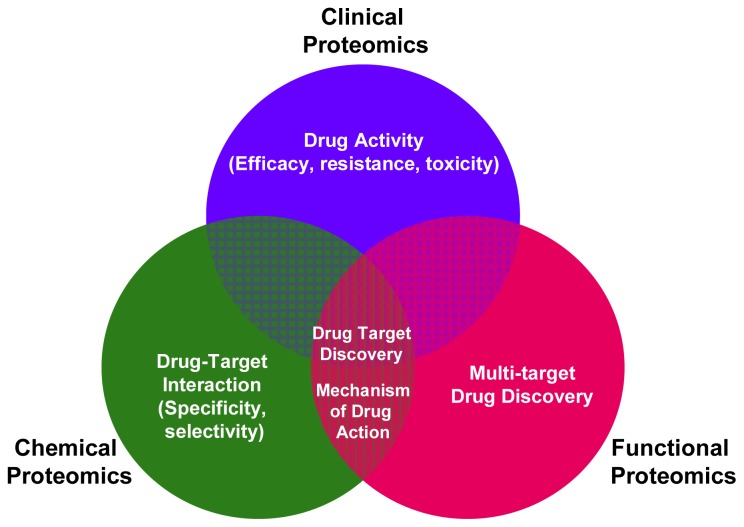
Main applications of functional, chemical and clinical proteomics in drug discovery.

The various MS-based platforms of clinical, functional and chemical proteomics can converge and create synergies in the modern process of drug discovery. As shown in Figure 1 the main contributions from functional, chemical and clinical proteomics to drug discovery development are underlined. In the complex drug discovery scenario, the various MS-based proteomic approaches extend beyond the common objective of drug target discovery, enabling the study of drug-target interaction (selectivity and specificity), drug activity (efficacy, resistance, toxicity) and elucidating the mechanism of action of a drug.

## 2. Applying Functional Proteomics to Biomarkers and Drug-Targets Discovery

The metabolism of a cell or of an entire organism is generally regulated by proteins, which act individually and, more frequently, in pathways. In particular, the function of a protein can be defined on the basis of its interactions whereas pathways are cascades of specific protein interactions that are necessary to activate distinct cellular functions [11]. Genetic mutations or environmental factors deregulate these pathways, leading to disease conditions. A detailed knowledge of the pathways active inside the cell and of how they are deranged in a particular pathology is fundamental for drug discovery as it allows the identification of new drug targets.

Functional proteomics focuses on the generation of information about proteins, such as expression levels, interacting partners, PTMs and activity, which all contribute to elucidate pathways active inside the cells and, ultimately, to a functional understanding of a biological system, including man. Before the advent of proteomics [12], classical biochemical studies delivered us the concept of mostly linear signal transduction pathway in which a signal (often extracellular) triggers the first member of the pathway, which in turn activates the second member and so on until the final member is activated, which is the effector of the biological function (for instance gene transcription, protein synthesis, regulation of metabolism, survival, proliferation, differentiation, migration and so on). In recent years, functional proteomics has been used to analyze not only the formation of specific protein-protein interaction, but also how these interactions lead to the assembly of macromolecular protein complexes that are regulated by PTMs and which affect pathway function. The emerging concept is that signaling pathways rely on protein interactions, which often do not form a linear string of events but instead distribute control through a network. In the analysis of such networks, the contribution of proteomics has been unparalleled. In fact, in mapping protein interaction networks and pathways by proteomic technologies, it was soon realized that the pathways and networks are also interconnected at many different levels [13]. On one hand, such cross-talk is of great biological importance, as it offers a means of generating functional redundancy, diversity and compensating mechanisms should parts of a pathway become unavailable. On the other hand, these findings are of utmost importance for the pharmaceutical industry, as they predict that it may be quite difficult to interrupt signaling networks by interventions targeting a single entity, thus furnishing an elegant molecular explanation to the clinical experience that the efficacy of cancer therapies based on a single drug is, with very few exceptions, quite limited [14]. This scenario also suggests a multi-target drug discovery therapeutic approach (Figure 1), which pharmaceutical companies considered so far counterintuitive, that is combining inhibitors of the first elements of a pathway (for instance a receptor) with inhibitors of downstream elements of the pathway (one or more of the effectors) to boost drug efficacy and prevent treatment resistance.

Functional proteomics has been widely applied to mapping signaling pathways in a number of pathologies and the vast literature published in the last ten years exceeds the scope of a single review. Therefore, only a few examples will be illustrated to describe some of the new paradigms in signaling networks that owe their discovery to proteomics.

The first step in defining a pathway or a network is to elucidate protein-protein interactions. Stable protein assemblies function as “molecular machines” in all cellular processes, from DNA replication and hnRNA splicing in the nucleus to protein degradation in the proteasome and ribosomal mRNA translation in the cytoplasm. The application of MS to this kind of biological problems was pioneered by the identification of the proteins of the yeast U1 snRNP in 1997 [15]. Since then the analysis of protein complexes has uncovered countless important global biological phenomena, including those which cause a pathological state when deregulated. Although proteomics has been very successful at determining the composition of complexes, the detailed study of binary protein interactions is still surprisingly difficult by proteomic methods. In part, this results from the general challenge of purifying protein pairs in the presence of other interacting proteins. As a result, binary protein interactions are still mostly identified by the yeast two-hybrid system, which has been already reviewed in a very clear manner for a readership not composed by molecular biologists [16]. Although this classical molecular biology approach has been automated to enable systematic proteomic scale studies of transient protein-protein interactions [17], the yeast two-hybrid system is not without issues as the interaction of two exogenous proteins in a yeast nucleus can lead to various artifacts. Proteomics technologies are more suited to study protein complexes by combining affinity tagging of proteins using genetic or molecular biology techniques and the speed and sensitivity of MS. Commonly used tags are the Flag peptide (DYKDDDDK or MDYKDDDDK), the Myc peptide (EEQKLISEEDL) [18] hemagglutinin, streptavidin, green fluorescent protein (GFP) and TAP (tandem affinity purification: a fusion cassette encoding calmodulin-binding peptide, a tobacco etch virus protease cleavage site and Protein A), and combinations thereof [19]. Tags allow efficient purification of stable protein complexes by sequential pulldown-elution-pulldown protocols [20]. The systematic application of these methodologies have enabled the definition of protein complexes in several model organisms among which the most thoroughly studied is yeast, whose proteome is organized in roughly 580 complexes each composed of 5–20 members [21–23]. Given the size of the complexes, it is rather obvious that each protein in the supramolecular structure cannot physically contact all the other proteins, and this is indeed the limit of the currently used proteomic pulldown experiments. In other words, we have all the components but no direct information on the interacting partner. The picture is further complicated by the fact that the structure of a complex can change dynamically over time, and certain complexes may only be necessary for a limited time to activate their downstream signal transduction pathways [14]. In order to study the dynamics of individual protein complexes other biochemical and cell biological techniques have to complement the proteomic approaches once the proteomic experiment has established the protein components of a complex [24], as exemplified in the definition of the TNFα/NF-κB signal transduction pathway described below.

The next level of cellular organization is provided by pathways and networks, in which proteins and protein complexes relay signals from the extracellular space into the cell or distribute information within a cell and its compartments. However, many more proteins are involved in networks than in typical protein complexes. One of the first human networks relevant for drug discovery defined with this approach was the TNF α/NF-κB signal transduction pathway [25]. It has long been known that the pro-inflammatory cytokine tumor necrosis factor (TNF)-α triggers a signaling cascade, converging on the activation of the transcription factor NF-κB, which forms the basis for numerous physiological and pathological processes. By using a combination of technologies from the fields of biochemistry (tandem affinity purification), proteomics (liquid-chromatography tandem mass spectrometry LC-MS/MS), bioinformatics (network analysis) and molecular biology (directed functional perturbation studies using RNA interference), 221 molecular associations and 80 previously unknown interactors were identified, including 10 new functional modulators of the pathway. The definition of such a network may facilitate the identification of new drug targets and to choose the most effective and specific intervention strategy [25]. Even more paradigmatic are the proteomic studies which defined in detail the epidermal growth factor receptor (EGFR) network, which is altered in various human cancers [14], and the breakpoint cluster region (BCR)-ABL1 network, responsible with few exception for the development of chronic myelogenous leukemia (CML) [26]. All these studies are summarized in an excellent, recent review [14], which clearly explains, among other examples, the molecular events at the basis of the clinical success of the BCR-ABL1 inhibitor imatinib (Gleevec, Novartis) [27] and suggests how functional proteomic studies can be used to determine drug action and predict potential side effects of the second-generation BCR-ABL1 inhibitor dasatinib (Sprycel; Bristol-Meyers Squibb) [14].

Studies in the field of functional proteomics led to the publication of hundreds of articles in the last few years, making an impressive amount of data available for drug discovery in a variety of pathological conditions, including liver cancer [28], hepatic injury [29], blood transfusion related diseases [30,31], neurodegenerative diseases [32], genetics diseases including gene therapy applications [33,34], endocrinology-related diseases [35] and male infertility [36,37]. A complete list of the diseases in which functional proteomics may boost drug discovery is outside of the scope of this review.

## 3. Applying Chemical Proteomics to Biomarkers and Drug-Targets Discovery

Chemical proteomics is a multidisciplinary research area integrating biochemistry and cell biology with organic synthesis and MS. The recent developments in affinity-based enrichment techniques in combination with MS have enabled the direct determination of protein binding profiles of small molecule drugs under more physiological conditions. In this context, chemical proteomics represents one of the most direct approaches to screen for drug–protein interactions [38,39]. One possible strategy typically involves the immobilization of a (chemically modified) drug to a solid-state support (for example, beads), either directly or by using a flexible linker. These functionalized beads are then incubated with a tissue extract or a cell lysate to allow proteins to bind to the drug. Finally, interacting proteins are eluted under either native or denaturing conditions, and they are digested and analyzed by MS.

The major drawback encountered in these affinity-based enrichment approaches is the presence in the pulled down extract of non specifically bound proteins. Among these, a large number of proteins which bind non-specifically to most conventional affinity matrix have been recently identified [40]. It is therefore strongly suggested to perform appropriate negative control experiments to distinguish nonspecific interactions from specific interactions.

The application of an inactivated affinity matrix and/or the preparation of an immobilized inactive drug derivative serves as an important factor in establishing whether proteins are non-specifically bound.

Another strategy is based on pretreatment of a cell lysate with free drug in a parallel experiment. This strategy allows the drug to interact with the target protein(s) before binding to the affinity matrix, so that comparative analysis of the parallel pulldowns reveals specific drug-binding proteins [41].

Such an approach becomes particularly powerful when combined with quantitative MS methods [39,42].

An example of the above-mentioned strategies which can be used to decrease the presence of non-specifically bound proteins is the recent identification of the interactome of the PDE5 inhibitors PF-4540124 [38], and PF-3717842 [43]. This approach shows how chemical proteomics might be used to profile the biochemical space (interactome) of small molecule inhibitors. Moreover, “compound-centric” chemical proteomics, as this approach also been called [44], allows the identification of binders of unexpected biochemical classes, including proteins without enzymatic function, and can therefore be used to find entirely novel targets [45–47].

A very relevant example of the strategy described above [47], was the assessment of the specificity profiles of the previously mentioned block busted drug BCR-ABL1 inhibitor imatinib and of two second-generation drugs: nilotinib and dastinib. These three drugs in a linkable version were analyzed for their target spectrum in affinity precipitation experiments. A very strong binding of immobilized imatinib to the quinone oxidoreductase NQO2 was detected, then confirmed by crystallographic studies. Instead, DDR1 and ARG were identified as novel interactors of the imatinib close analog nilotinib. Finally, dasatinib (Sprycel; Bristol-Meyers Squibb), originally developed as a dual specificity ABL- and SCR-family kinase inhibitor, was found to interact with up to 24 protein kinases [10]. Considering the market share of these three drugs, the studies reported above and in the previous section [14,27] are certainly major findings of proteomics in drug studies.

Another important tool in the field of chemical proteomics is based on carefully designed chemical probes, also defined “activity-based probes”, that can specifically target diverse sets of enzyme families and provides direct information about the activation state of identified proteins. A chemical probe contains three parts, a reactive ligand that can covalently bind to the target protein/enzyme, a linker region modulating the reactivity and specificity of the reactive ligand, and a tag for identification and purification of the target protein/enzyme. Only active enzymes of the targeted family will react with the ligand and will therefore be identified [48].

This method can lead to the identification of new proteins with the respective biochemical activity, thus allowing the identification of new drug targets. Alternatively, it can be applied to determine the selectivity profile of drugs targeting an enzyme family. This can be done via pre-treatment of the lysate with the drug of interest (which prevents the reactive ligand of the probe from covalently binding the drug-interacting enzyme) and subsequent labeling and identifying of the remaining enzymes using appropriate reactive probes.

Several kinds of chemical probes have been used in proteomic studies, for a multitude of enzyme classes such as hydrolases, proteases, kinases, phosphatases, histone deacetyilases, glycosidases and oxidoreductases [49].

Therefore, chemical proteomics plays a significant role in drug discovery as it becomes possible not only to identify new drug targets, but also to profile the selectivity of drugs and their mechanisms of action systematically in relevant tissues (Figure 1).

In particular, the identification of all the proteins which interact with a given drug in a cell or tissue might help predict or elucidate the side effects of that drug, which is of fundamental interest for the pharmaceutical companies.

Excellent recent reviews on comprehensive potential applications of chemical proteomics in the drug discovery process are available [10,44,50].

As an example of a chemical proteomics approach which can give an incredible input to targeted-drug discovery we report chemical proteomic strategies for the enrichment and quantification of the accessible vascular proteome. In fact, an important challenge for novel targets discovery is to identify drug targets which are readily accessible to treatment in a diseased tissue (for example tumoral tissue).

Due to the poor selectivity of most conventional pharmaceuticals used in cancer therapy, “ligand-based vascular targeting” strategies open the horizon towards the development of more selective and better tolerated anti-cancer drugs.

In this context, the “*in vivo*/*ex vivo* biotinylation technique” represents an elegant method for the identification of proteins which are readily accessible from the vasculature. This technique relies on the perfusion of animals [51,52] or surgically resected organs [53,54] with reactive ester derivatives of biotin. Biotinylated proteins are recovered from normal and tumor tissues by capture on streptavidin-sepharose, after lysis in presence of strong detergents. After on-resin digestion of biotinylated protein, resulting peptides are subjected to comparative proteomic analysis, which allows the identification and the relative quantification of vascular proteins in normal organs and in tumors, thus making the discovery of accessible novel drug targets possible [55,56]. Other strategies suitable for the discovery of vascular-accessible tumor markers such as “cell surface capturing” and “silica coating” have been extensively and recently reviewed [57].

## 4. Applying Clinical Proteomics to Biomarkers and Drug-Targets Discovery

**Figure 2:**
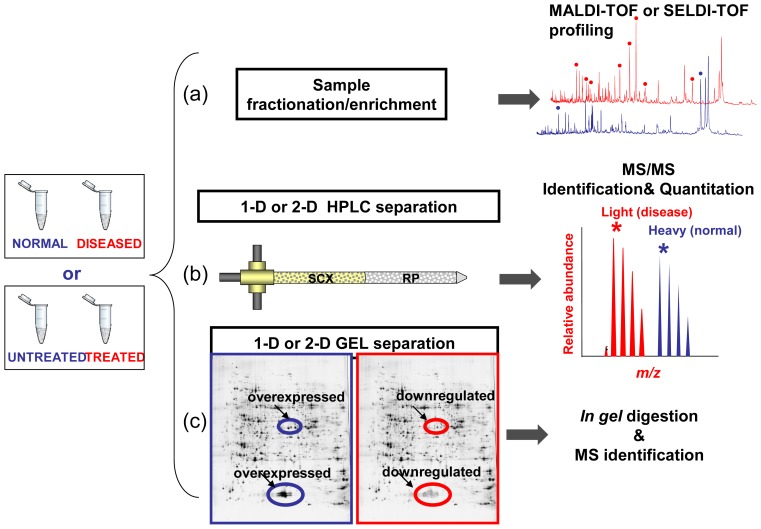
Biomarker discovery approaches in clinical proteomics. Samples (normal *vs.* diseased or drug-treated *vs.* untreated) are fractionated and then Mass Spectrometry (MS)-analyzed according to three main strategies. (**a**) Molecular profiling aimed to the generation and comparative analysis of fingerprints between healthy/diseased or drug-treated/untreated biospecimen. (**b**) High Performance Liquid Chromatography coupled to Mass Spectrometry (LC-MS/MS) for both quantitation and identification of protein. (**c**) Mono-or bi-dimensional Gel Electrophoresis (1DE or 2DE) followed by identification of bands or spots in which quantitative variations of protein expression are observed.

The proteome records the flow of information that starting within the cells, through the intercellular protein network, goes beyond the extracellular microenviroment up to come to the blood macroenviroment [58]. Accordingly, the proteome may reflect immediate and characteristic changes in response to disease processes and external stimulation. Like the proteome, the proteolytic degradation products of the proteome, the so-called low-molecular-weight (LMW) proteome, or peptidome, may also have the potential to contain disease-specific information [59,60]. The peptidome is also referred to endogenous peptides which have very specific functions as mediators and indicators of biological processes, which play important roles as messengers, e.g., as hormones, growth factors, and cytokines, and thus have a high impact on health and diseases [61]. By the means of proteomic tools such as MS, it is possible to qualitatively and quantitatively reveal molecular profiles contained in healthy or clinical samples. Consequently, MS technologies offer the opportunity to screen and discovery simultaneously multiple biomarkers, which consist of a pattern of up- or down-regulated molecules (proteins, peptides, metabolites, organic molecules) representative of a given (healthy/disease) condition. Basically, clinical proteomics covers all MS-based preclinical and basic science studies aimed at discovering and understanding the role of proteins in pathological processes in order to facilitate the early diagnosis of disease, the prognosis prediction, the identification of new therapeutic targets and the evaluation of treatment response [6,58,62]. So the application of proteomics tools in the field of medicine may accelerate the understanding of diseases and may facilitate the discovery of new drug targets and diagnostic markers. As outlined in Figure 2 the MS-based biomarker discovery in clinical proteomics could be addressed by three main approaches: (i) comparative analysis of molecular signatures from enriched or fractionated subproteomes from healthy/diseased or drug untreated/treated bio-samples by Matrix-Assisted Laser Desorption/Ionization Time-Of-Flight Mass Spectrometry (MALDI-TOF) or Surface-Enhanced Laser Desorption/Ionization time-of-flight mass spectrometry (SELDI-TOF MS); (ii) High Performance Liquid Chromatography coupled to electrospray ionization (ESI) tandem Mass Spectrometry (MS/MS) for both quantitation and identification of protein found differently expressed between healthy/diseased or drug untreated/treated samples; (iii) Mono-or bi-dimensional gel electrophoresis (1DE or 2DE) followed by identification by MALDI-TOF MS or LC-MS/MS of bands or spots in which quantitative variations of protein expression are observed between healthy/diseased or drug-treated/untreated samples. All the above mentioned state-of-art strategies are those commonly followed in the current clinical proteomic studies even if the rapidly evolving MS and proteomic-related technologies are able to provide new forthcoming platforms which can offer novel “modus operandi” in this field [63,64].

The first approach was pioneered by Liotta and Petricoin [65] by SELDI-TOF MS-based pattern recognition in protein profiles from serum; despite initial debates and controversies [66,67], this approach has then gathered great interest and consensus in the proteomic community [68–74]. So one promising field of clinical proteomics is nowadays represented by SELDI and more importantly MALDI-TOF MS-based approaches for profiling clinical samples. This strategy may appear as a direct avenue for biomarker discovery because it consists in the direct analysis of low molecular weight metabolites, endogenous peptides and proteins in readily accessible bodily fluids or, more difficult to obtain, in sample tissues. However, the complexity and high dynamic range of such biological samples (plasma, serum, induced sputum, saliva, synovial fluid and so on) make the characterization of metabolites as well as endogenous peptides and proteins a challenging task. Salts and abundant protein components (e.g., albumin in serum or plasma; mucins in induced sputum, hyaluronic acid polymers in synovial fluid) typically interfere with proteomic, peptidomic and metabolomic analysis. As a consequence, a sample pretreatment is required before MS analysis. In SELDI-TOF MS this issue is addressed by sample fractionation on a chip covered by specific chromatographic surfaces. Proteins from row samples are allowed to bind to the surface, which is then washed to remove salts and unbound species and the corresponding protein profile is then generated by the mass spectrometer directly from the chip [75]. When MALDI-TOF is used, the pre-fractionation has to be performed separately. The off-line fractionation and/or enrichment of the proteome of clinical sample before MALDI-TOF MS analysis may be pursued by different procedures. In concomitance to well-known magnetic beads [76–80], or classical solid phase extraction procedures [81], innovative platforms and devices for selective capture of peptides, phosphopeptides and polypeptides from bodily fluids and tissues, have been recently proved to be very successful [61,82,83]. Despite its unquestionable throughput, SELDI-TOF does not offer the possibility to independently optimize sample protocols or preparation aimed at reducing complexity or enriching more in depth a particular sub-proteome; these possibilities are instead granted by the MALDI-TOF technology.

Panels of peptides of potential diagnostic importance present in blood plasma/serum have been produced for diseases such as gastric cancer [84], ovarian cancer [85], colorectal cancer [86], Alzheimer’s disease [87], oral cancer [88], nasopharyngeal tumor [89], asthma [77], brain tumor [90], multiple sclerosis [91] and renal cell carcinoma [92]. Furthermore, to give such an example of potential markers of pathology found in other bodily fluids such as urine, we can cite the recent study by Maini *et al.*[93] on high altitude hypoxia as a model of the pathophysiology of diseases related to tissue hypoxia. Other potential peptide biomarkers of chronic obstructive pulmonary disease have found in bronchoalveolar lavage fluid [94] or in induced sputum [95].

In the second approach, the classical “bottom-up” procedure, digested peptides of protein extracts from bio-specimen are fractionated by mono-dimensional (reverse phase) or bi-dimensional (strong-cation-exchange followed by reverse phase) LC and then identified by MS/MS. By the mean of differential isotopic labeling procedure of the samples it is also possible to accurately quantify differential protein expression in different samples (in clinical proteomics healthy/disease or drug-treated/untreated).

ICAT (Isotope Coded Affinity Tags) [96], iTRAQ *(*isobaric Tags for Relative and Absolute Quantitation) [97] and SILAC (stable isotope labeling by amino acids in cell culture) are the acronyms of most common MS accurate quantitative strategies used in proteomic workflows for biomarker discovery [98]. Depending by the nature of labeling, MS-based quantitation may be performed at MS (signals differing by a specific *m/z* shift depending by the isotopic labeling such as ICAT or SILAC) or at MS/MS level (isobaric labeling such as iTRAQ). For instance, in SILAC quantitation is performed in full MS and identification in MS/MS, while in iTRAQ the identification and quantitation are performed in MS/MS. An excellent example of application of these labeling quantitative approaches is given by a recent study on differential proteins expression in the proteome of vascular smooth muscle cells (A7r5) when treated with individual (*R*) or (*S*) enantiomers of the beta(1)-adrenergic antagonist, atenolol. This investigation was assessed by iTRAQ-coupled to two-dimensional LC-MS/MS, revealing that some calcium-binding proteins were down-regulated and a series of enzymes involved NAD+/NADH metabolism were up-regulated in cells incubated with the *S*-enantiomer relative to the cells incubated with the *R*-enantiomer. The Authors also demonstrated lower intracellular Ca^2+^ concentration and a higher ratio of NAD+/NADH in A7r5 cells incubated with the *S*-enantiomer of atenolol. As Ca^2+^ signals transduced by calcium-binding proteins act on cytoskeletal proteins, which play a fundamental role in the regulation of the cytoskeletal modeling, the results presented provide a potential molecular explanation of the therapeutic effects of atenolol [99]. The above-cited study also highlights the expanding contribution of proteomics in assessing the role of chirality in drug action. For a more extensive description of this topic we recommend a recent review by Sui *et al.*[100].

Although labeling experiments gain a precise quantification [101], these MS-quantitative strategies increase the costs and the complexity of the entire experiment and, as a consequence, the low sample throughput is a great limitation for clinical proteomics studies. In this direction, the so-called “label-free” strategies [102] appear to be more effective for large-scale biomarker discovery in complex samples. The examples reported below demonstrate not only the efforts to build bridges between high-performance proteomics and clinical routine, but also emphasize how discovery-based proteomics analyses of clinical samples allow the identification of new potential biomarkers and therapeutic targets in different kind of diseases.

A label free approach based on nano-LC/MS was performed by Nanni *et al.* for quantitative serum peptidomics analysis in Crohn’s disease patients [103]. In another study protein extracts from normal lung and non-small cell lung cancer tissues were digested and subsequently enriched in phosphopeptide content. LC-MS/MS, label-free quantitative and bioinformatic analysis provided dozens of differential markers able to discriminate between normal and tumor tissue [104]. Very recently, researchers from the Fred Hutchinson Cancer Research Center have applied the “label-free” technology in order to distinguish, on a quantitative-proteomic basis, known classes of leukemias: acute myeloid leukemia (AML) and acute lymphoid leukemia (ALL). Proteins were extracted from blasts derived from four patients with AML, five patients with ALL and, as control, from CD34^+^ cells purified from six healthy donors and mononuclear cells (MNC) from two healthy donors. Proteins were analyzed by LC-MS/MS and quantified with a label-free alignment-based algorithm developed in the Author’s laboratory. The four proteins best able to distinguish CD34^+^, AML, and ALL were all either known biomarkers or proteins whose biological functions are consistent with their ability to distinguish the classes examined. So, this approach could represent a very promising strategy for classification of different classes of pathology [105].

Less traditional but more exclusive is the use of the “top-down” procedure in which, contrary to the “bottom-up” strategy, it is possible to analyze intact proteins (which are not enzymatically digested prior to MS) by the mean of FT-ICR MS (Fourier transform-ion cyclotron resonance) which achieves mass resolution (100,000) and a mass accuracy (1 ppm) superior than other MS platform. In this case this platform is well suited for detection of protein PTMs [106] which are not easily detected by standard protein profiling techniques.

The third approach is outlined in Figure 2 panel (c). Differently expressed bands from 1-D or spots from 2-D gels in the two state (disease/normal) are excised and than the proteins are *in-gel* digested and the resulting peptides are identified by MS. Although less used than the other two approaches described above, 1-D and 2-D GE have recently given an important contribution in the field of biomarker discovery. In the case of 1-D GE we report the work of Berardi *et al.* in which four proteins, found to be overexpressed in endothelial cells (ECs) of patients with active MM (MMECs), were *in-gel* digested and identified by MS as filamin A, vimentin, α-crystallin B and 14-3-3ζ/δ protein, not yet linked to overangiogenic phenotype. Further analyses demonstrated the role of the newly identified proteins in the overangiogenic phenotype of MMECs, suggesting that they could be new targets for the antiangiogenic management of MM patients [107].

In a retrospective study focused on a population of patients with ductal pN0M0 tumors, a 2D-electrophoresis (2-DE) proteomic approach was used to identify novel biomarkers in node-negative breast cancers. A subset of 20 patients who developed metastases were compared to another subset (21 patients) in whom no metastatic relapse took place. The differential analysis of more than 2000 spots in 2-DE gels allowed the identification of 13 differentially expressed proteins, which were confirmed by western blotting. In the subset of patients who developed metastases, two proteins, GPDA and FABP4, were down-regulated whereas all the others were up-regulated [108]. A recent improvement in 2-DE technology has been achieved in 2D-DIGE (differential in gel electrophoresis) which substantially increases gel reproducibility allowing a better quantitative analysis by the use of fluorescent dyes. Proteins from different disease states are labeled with different dyes then the samples are combined and protein separated by 2DE. The gel is finally scanned at different wavelengths thus generating different images. The quantitative analysis of differentially expressed spots is performed by the superimposition of the images using specialized software packages. In order to search for identification of differentially expressed protein in renal cell carcinoma (RCC), the most common neoplasm affecting the adult kidney, by a 2-D DIGE followed by MS, 20 RCC tissues were compared with matched adjacent normal kidney cortex (ANK). After gel analysis, 2500 differentially expressed protein spots were excised and a total of 100 proteins were identified by MS, 23 and 77 of which were, respectively, over- and down-regulated in RCC as compared to ANK. The DIGE data were also confirmed by immunoblotting for the identified proteins [109].

### Limitations of Comparative Profiling Strategies

It is now worth highlighting some points which must be careful considered in clinical proteomics studies. Many factors may affect the reproducibility of data generated and extracted from mass spectra (*m/z*, peak intensity, peak area) which are used for discriminatory pattern recognition between normal *vs.* diseased or drug-treated sample. First of all, variability may affect clinical sample collection and handling: an example is given by blood processing for serum or plasma, or the use of protease inhibitor cocktails, or storage conditions applied to a date clinical sample [110]. Another source of variability resides in sample preparation and/or fractionation and in all pre-analytical steps and enrichment strategies used in combination with MS-analysis [111,112]. Remarkably, the MALDI-matrix application and the instrumental settings as well as the bioinformatics analysis could all be source of variable fluctuations [113]. In addition to the above listed technical limitations, it is also important to consider the patients/controls individual variability. Since a variety of factors may influence each individual profile, we cannot assume that drug responses will be similar in healthy or diseased group [114]. Another important factor which cannot be underestimated is the time between drug treatment/administration and analysis. In fact if the drug does not reach the pharmacokinetic steady state, the protein profiles changes may not correlate to the drug treatment/administration [115]. Therefore, an in depth assessment of proteomic variability is mandatory for a better understanding and data interpretation of clinical proteomics studies. In this direction, procedures for quality control of proteome profiles have been recently suggested [116]. Additionally, recommendations concerning minimal information about a proteomic experiment have been released by the Human Proteome Organization in order to increase the independent reproducibility of published data [117]. One of the limitations of biomarkers and drug-target discovery studies performed so far is that very often the putative biomarker/drug-target was searched in a single tissue/body fluid. This was due to the limited output of the classical biochemical strategies. The high-throughput, which is made available by the proteomics approaches, now allows a multi-tissue/body fluids research strategy. In fact, by searching for a biomarker/drug-target in multiple biological samples, both systemic (serum, plasma, urine), proximal (induced sputum, nipple aspirate, saliva, synovial fluid, *etc.*) and in situ (target tissue), it should be possible to maximize the success rate. Unfortunately, few studies [118–120] of this kind have been published so far; however, it may be expected that many more will appear in the next future. Systematic review of the published results will then help pharmaceutical companies and clinicians in the decision making process.

Last but not least, individual variability may constitute a big hurdle in MS-based disease biomarker discovery, in particular for pathologies with multifactorial etiology such as cardiovascular disease, cancer or diabetes, leading to false discovery when small patient cohorts are assayed. In fact, differential protein expression detected by proteomics approaches may be due to the interindividual variability in protein abundance rather to the pathology examined. Therefore, in order to overcome this problem, it is imperative to work with a large number of patients and the putative markers arising from the discovery phase have to be validated by specific and quantitative measurements [121].

The validation of candidate biomarkers is a critical issue in the discovery pipeline. In fact, if on one hand we are witnessing a constant increase of the number of the putative MS-based biomarkers, on the other hand, a limited number of specific antibodies are currently available in the traditional antibody-based approaches used for validation. However, among the new emergent MS-based validation assays, stable isotope dilution-multiple reaction monitoring (MRM) [122] and stable isotope standards with capture by anti-peptide antibodies (SISCAPA) [123] seem to address the need for a higher throughput of biomarker validation. MRM-MS for protein assays is based on measurement of tryptic (proteotypic) peptides of the protein/biomarker candidates. In particular the selection of 3–5 peptides per protein is performed and the corresponding synthesized stable isotope-labeled peptides are used as internal standards. Specific fragment-ion signals derived from the endogenous unlabeled species are compared to those from the exogenous labeled peptides and from the ratio of the peak area it is possible to obtain an accurate measure of the concentration of the corresponding protein.

The SISCAPA assay is based on the use of antipeptide antibodies against the selected proteotypic peptides from the proteins of interest. Indeed, antipeptide antibodies suitable for the SISCAPA assay are much easier to obtain as compared to equivalent antibodies directed against the corresponding full-length proteins [121]. The method utilizes antibody-nanoaffinity columns to enrich for these specific peptides, as well as stable-isotope-labeled internal standards of the same sequence, which have been spiked into the sample. The relative amounts of stable isotope labeled standard peptide added to the sample and the selected peptide are measured by MRM. Thus, the MS serves as the second antibody. Due to its multiplexing capability, MRM is ideal for high-throughput multiplexed quantification of a significant number of proteins. In fact, this methodology allows to quantify more than 100 different proteins in a single MRM LC-MS run [124]. However, without depletion of high abundance proteins and prefractionation, LC-MRM-based assays are generally only able to quantify moderate-abundance proteins in the low microgramme per milliliter concentration range using conventional platforms [125]. For this reason, many efforts, recently and extensively reviewed by Shi and colleagues, have been done to address the low sensitivity issue in MRM-based assays [126]. It is worth mentioning the limit of quantitation in the 50–100 pg/mL range achieved by using a magnetic bead-based platform for high throughput sample processing implemented in a multiplexed SISCAPA assay [127]. Also, recent developments in MS technology (outside the scope of this review) such as the use of ion funnels [128] or the “field asymmetric waveform ion mobility” MS [129], by providing an higher signal intensity and resolving power respectively, may increase MRM sensitivity. Other progress in sample prefractionation/enrichment strategies as well as advances in MS instrumentation could allow to MRM-based assays to become the golden gate which links biomarker discovery to clinical use.

## 5. Conclusions

The escalation of MS technologies and related functional, chemical and clinical proteomics approaches could greatly impact the biomarker and drug discovery process.

Functional proteomics approaches explore and elucidate the components of pathways and their interactions which, when deregulated, lead to a disease condition. The detailed knowledge of how pathways and networks are deranged may help in identifying new drug-targets.

Chemical proteomics strategies aiming at the comprehension of drug interactome can contribute to optimization of lead compounds improving drug selectivity and specificity, thus reducing side effects.

The application of MS-based proteomics to the study of human diseases and their translation to the clinic has opened new horizons for medicine.

The comparison of protein expression between healthy individuals and patients is one of the most promising tools for biomarker identification. The main requirements for a platform to be used in clinical proteomic studies aiming at biomarker discovery are: sensitivity, accuracy, reproducibility and high sample throughput. Moreover, it must provide extensive proteome coverage of complex samples in order to allow the detection of proteins whose concentration levels vary by several orders of magnitude. So far, many proteomic technologies have been employed in the field of protein biomarker discovery. High-throughput MS-based profiling strategies for clinical samples such as tissue and bodily fluids present a direct avenue for biomarker discovery. MALDI-TOF and SELDI have recently captured significant attention for the rapid determination of molecular masses and the ability of screening with high-throughput small amounts of clinical samples.

Biomarker’s long journey from the bench to the clinic is at its beginnings, but the rapidly developing MS-based proteomics technology could accelerate this process.

The sensitivity of modern mass spectrometers strongly depends on the type of instrument and it is constantly improving with time. Nevertheless, improving selectivity of the analysis rather than MS sensitivity could possibly be an additional way of looking into low-abundance species. In this respect, the possibility to develop devices allowing selective isolation of LMW peptides prior to MS analysis may accelerate the discovery rate of potential biomarkers and drug targets.
